# Photocatalytic Degradation of Azithromycin by Nanostructured TiO_2_ Film: Kinetics, Degradation Products, and Toxicity

**DOI:** 10.3390/ma12060873

**Published:** 2019-03-15

**Authors:** Mirta Čizmić, Davor Ljubas, Marko Rožman, Danijela Ašperger, Lidija Ćurković, Sandra Babić

**Affiliations:** 1 Department of Analytical Chemistry, Faculty of Chemical Engineering and Technology, University of Zagreb, 10000 Zagreb, Croatia; diva@fkit.hr (D.A.); sbabic@fkit.hr (S.B.); 2 Department of Energy, Power Engineering and Environment, Faculty of Mechanical Engineering and Naval Architecture, University of Zagreb, 10000 Zagreb, Croatia; davor.ljubas@fsb.hr; 3 Laboratory for Mass Spectrometry, Division of Physical Chemistry, Ruđer Bošković Institute, 10000 Zagreb, Croatia; marko.rozman@irb.hr; 4 Department of Materials, Faculty of Mechanical Engineering and Naval Architecture, University of Zagreb, 10000 Zagreb, Croatia; lidija.curkovic@fsb.hr

**Keywords:** azithromycin, photocatalysis, UV mercury lamp, LED lamp, *Vibrio fischeri* toxicity

## Abstract

In this paper, nanostructured TiO_2_ film was prepared by the by sol-gel process and dip-coating technique with titanium tetraisopropoxide as a precursor. After heat treatment at 550 °C, the deposited film was characterized by means of micro-Raman spectroscopy and atomic force microscopy (AFM). It was found that the TiO_2_ film consisted of only the TiO_2_ anatase phase and showed a granular microstructure. Photocatalytic degradation of azithromycin by using sol-gel nanostructured TiO_2_ film was studied to define the most effective degradation process for potential use in wastewater treatment. Different factors were evaluated during photocatalysis, such as pH (3, 7, and 10), water matrix (ultrapure water and synthetic municipal waste water effluent), influence of another pharmaceutically active compound (sulfamethoxazole, one of the most often detected pharmaceutic compounds in waste waters), and radiation sources (low pressure ultraviolet (UV) mercury lamps with a UV-A and UV-C range; a light-emitting diode (LED) lamp with a radiation peak at 365 nm). The most effective degradation process was achieved with the UV-C irradiation source in matrices at pH 10. The water matrix had little effect on the photocatalytic degradation rates of azithromycin. The presence of sulfamethoxazole in the water matrix decreased the degradation rate of azithromycin, however, only in matrices with a pH level adjusted to 10. During the experiments, five azithromycin degradation products were identified and none of them showed toxic properties, suggesting effective removal of azithromycin. LED 365 nm as the irradiation source was not as effective as the UV-C lamp. Nevertheless, considering the cost, energy efficiency, and environmental aspects of the irradiation source, the LED lamp could be a “real-life” alternative.

## 1. Introduction

The presence of pharmaceuticals in the environment has been the focus of scientific research world-wide for several decades and the issue it poses for the environment is well established. Pharmaceuticals may reach the environment via different pathways, but wastewater treatment plants are considered to be prominent [1]. Conventional wastewater treatment plants are not designed for the purpose of removing complex organic compounds, such as pharmaceuticals, their metabolites, and potential transformation products. Therefore, these compounds reach the environment without being decomposed during treatment. Once they reach the environment, they may have possible negative effects on all types of organisms. They influence organisms they encounter and the resulting consequences are still under investigation. To control their levels in the environment, and in that way, minimize possible negative effects, legal definitions of their concentrations in the environment are required. The European Union issued Decision 2015/495/EU, which deals with compounds that may have possible negative influences on the environment. However, aggravating circumstances exist, including the lack of data on the chronic toxicity of such compounds, and ecotoxicological data for mixtures of pharmaceuticals, their metabolites, and transformation products [2]. Some studies reported that microorganisms that were once prone to antibiotics are now showing resistance and therefore pose a global threat to human health [3,4]. Other adverse properties that pharmaceuticals may show in the environment are toxicity and phytotoxicity. The combination of all the mentioned properties can result in an imbalance in the complete ecosystem [3].

Considering that conventional waste water treatment processes are ineffective in the removal of complex organic compounds, such as pharmaceuticals and their related compounds, alternative methods of water treatment are necessary [1]. Advanced oxidation processes (AOPs) are considered alternative wastewater treatment processes that can be implemented in conventional treatment plants to improve their efficiency. AOPs are based on the formation of very reactive species that are unselective and with enough potential to degrade complex organic compounds. 

One of the AOPs is photocatalytic oxidation. A photocatalytic system consists of a photocatalyst of choice together with an appropriate irradiation source. Photocatalysis in general is applicable to the degradation of organic pollutants, such as pharmaceuticals [5]. Among the semiconductor catalysts, titanium dioxide (titania, TiO_2_) is the most frequently used in the field of photocatalysis due to its exceptional chemical and physical properties. TiO_2_ crystal structures can be distinguished in three polymorphs: Rutile (tetragonal), anatase (tetragonal), and brookite (orthorhombic). Among these, the anatase phase usually exhibits the best photocatalytic behavior [6]. 

As an irradiation source, UV light is often used because the energy provided with this source is enough to activate the photocatalytic reaction. LED lamps are introduced as an alternative light source to mercury based sources because of their numerous advantages. 

In this work, azithromycin and sulfamethoxazole were selected because of their low removal rate during conventional waste water treatments [3,4,7,8,9] and their frequent occurrence in the environment [8,9,10]. Some research reports that sulfamethoxazole can affect non-target organisms at environmentally relevant concentrations (μg/L) [11]. Sulfonamides, the antibiotic group of sulfamethoxazole, are present in the environment and are highly toxic to microorganisms, algae, and certain plants, but higher risk is caused by the generation of drug resistance [4]. Azithromycin is a macrolide antibiotic and this group of antibiotics also exhibits toxicity [2]. Based on the gathered data on azithromycin’s appearance in the environment, its low removal rate, and toxicity, it was included in the watch list of the European Union, defined in the Decision 2015/495/EU [7].

In this work, nanostructured TiO_2_ film was deposited on a borosilicate glass substrate by the sol-gel process and dip coating method. Antibiotic azithromycin was subjected to photocatalytic degradation using sol-gel nanostructured TiO_2_ film and different radiation sources (low pressure Hg based UV-C and UV-A lamps and UV-A LED lamp). To analyze the microstructure and morphology of the deposited nanostructured TiO_2_ film, micro-Raman spectroscopy and atomic force microscopy (AFM) analyses were performed. The process was optimized in terms of the pH, water matrix, and the influence of another compound (sulfamethoxazole, a widely used antibiotic). Also, degradation products that occurred during the degradation period were defined and monitored using liquid chromatography in tandem with mass spectrometry. Toxicity with *Vibrio fischeri* was determined for samples after photocatalytic degradation to define the efficiency of the degradation. 

The aim of this research was to contribute to defining and understanding the optimal photocatalytic degradation of azithromycin, an antibiotic of great environmental concern.

## 2. Materials and Methods 

### 2.1. Chemicals and Reagents

Azithromycin (AZI) was received from Veterina Animal Health (Kalinovica, Croatia) and sulfamethoxazole (SMETOX) from Sigma Aldirch (St. Louis, MO, USA). Both were analytical grade standards with purity >98%. All experiments were performed in ultrapure water prepared by a Millipore Simplicity UV system (Millipore Corporation, Billerica, MA, USA). Pharmaceuticals were prepared at a concentration of 10 mg/L for all experiments. For the chromatographic analysis, HPLC grade solvents were used: Methanol (J. T. Baker, Deventer, Netherlands) and acetonitrile (Fischer Scientific, Leicester, UK). pH adjustment was done using sodium hydroxide (0.01 mol/L) or hydrochloric acid (0.1 mol/L). Synthetic municipal waste water treatment plant effluent (SE) was prepared according to the OECD (The Organisation for Economic Co-operation and Development) Guidelines for Testing Chemicals: 32 mg/L peptone, 22 mg/L meat extract, 6 mg/L urea, 28 mg/L K_2_HPO_4_, 4 mg/L CaCl_2_ × 2H_2_O, 7 mg/L NaCl, and 2 mg/L Mg_2_SO_4_ × 7H_2_O.

For the preparation of TiO_2_ sol (colloidal solution), the following components were used: Titanium (IV) isopropoxide (Ti(C_3_H_5_O_12_)_4_)-TIP as a precursor, i-propanol (C_3_H_7_OH)-PrOH as a solvent, acetylacetone (CH_3_(CO)CH_2_(CO)CH_3_)-AcAc as a chelating agent, nitric acid (HNO_3_)-HN-0.5 M as a catalyst, and polyethylene glycol (H(OCH_2_CH_2_)_n_OH), Mr = 5000–7000, as an organic/polymer additive in the amount of 2 g/100 mL. All these chemicals were analytical grade reagents. The molar ratio of these reactants was: TIP:PrOH:AcAc:HN = 1:35:0.63:0.015. 

For the toxicity experiments, the bacterial assay was a commercially available system, LUMIStox 300, with a water bath LUMIStherm luminescent bacteria test, LCK 484, with reactivation solution (Hach Lange, Varaždin, Croatia).

### 2.2. Characterization of Nanostructured TiO_2_ Film

For characterization of the nanostructured TiO_2_ film, micro-Raman and atomic force microsopy (AFM, Multimode AFM with a Nanoscope IIIa controller (Veeco Instruments, Santa Barbara, CA, USA)) analyses were used. For this purpose, the nanostructured TiO_2_ film was deposited on a borosilicate glass plate under the same conditions and procedure as well as for the deposition of the catalyst on the both reactors.

Micro-Raman analyses were performed using a Bruker SENTERRA Dispersive Raman spectrometer (Billerica, MA, USA) equipped with an Olympus microscope. The Raman spectra of the nanostructured TiO_2_ film was analyzed at randomly selected points and under the same conditions—diode laser (AlGaAs) operating at 785 nm, nominal laser power of 100 mW, excitation power of 10 mW, magnification of 50×, aperture 25 × 1000 µm, spectral resolution of 3–5 cm^−1^, grating of 1200a, 20 scans, and 5 s of integration time. Spectra were recorded in the frequency range of 50–1350 cm^−1^, and as a detector, a Peltier-cooled charge coupled device (CCD) camera was used.

The surface topography of the nanostructured TiO_2_ film was determined by using a Multimode AFM with a Nanoscope IIIa controller (Veeco Instruments Santa Barbara, CA, USA) with a vertical engagement 125 μm scanner (JV). Contact mode imaging was performed under ambient conditions in air by using silicon tips (NP, Nom. Freq. 18 kHz, Nom. Spring constant of 0.06 N/m), at a scan resolution of 512 samples per line. The linear scanning rate was optimized between 1.0 and 2.0 Hz at a scan angle of 0°. Images were processed and analyzed by means of the offline AFM NanoScope software, version 5.12r5. Particle dimensions of the granular microstructure of the TiO_2_ film were determined by the Particle Analysis option within the AFM software. 

### 2.3. Photolytic and Photocatalytic Experiment Setup

The catalyst in all experiments was TiO_2_ in the form of three layers deposited on the borosilicate glass substrate using the sol-gel method and dip-coating technique. The film was dried at 100 °C for 1 h prior to the deposition of the next layer. After the deposition of the layers, the deposited film was heat-treated at 550 °C for 4 h [6]. 

In the photocatalytic experiments, two reactor types were used:

- Reactor with Pen-Ray model lamps (Reactor 1), where the catalyst was deposited on reactor inner wall; and
- reactor with an LED lamp (Hönle Group UVA HAND LED 365) (Reactor 2), where the catalyst was deposited on the glass ring that was placed at the bottom of the reactor.

Reactor configurations differed because one of the aims was to achieve optimal irradiation of the catalyst. Pen-Ray lamps, because of their shape, better irradiate the catalyst when it is placed on the reactor wall and the LED lamp achieves better irradiation when the catalyst is placed on the plate at the bottom of the reactor.

**Figure 1:**
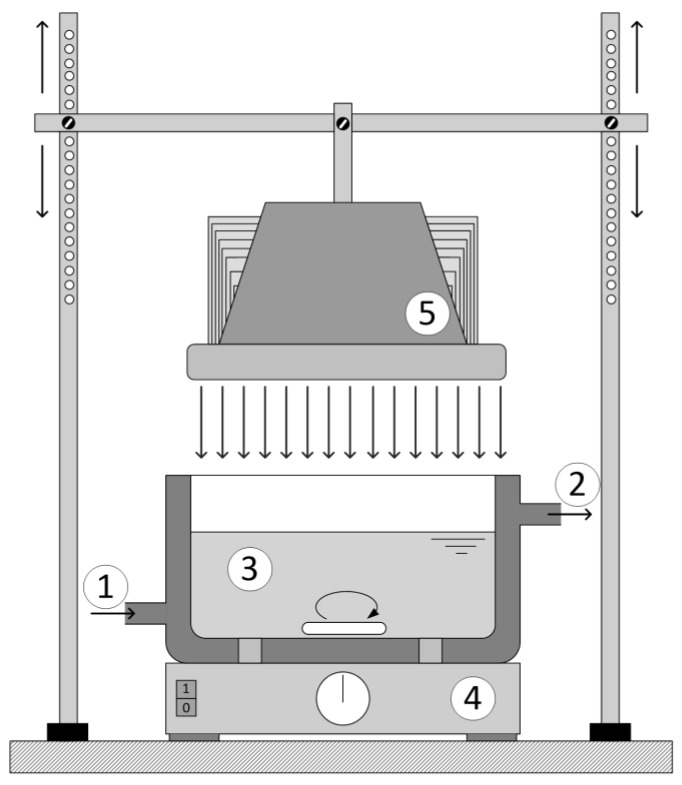
Experimental system for experiments with the LED lamp: 1—cooling water-in, 2—cooling water-out, 3—irradiated solution, 4—magnetic mixer, 5—LED lamp.

Reactor 1 was cylindrical in shape was 0.11 L (Figure 1). The reactor was kept under a constant temperature of 25 ± 0.2 °C and with continuous purging with air. The light sources used were low pressure Hg lamps with a predominant range in UV-A (365 nm) and UV-C (254/185 nm). The irradiation source of the UV-A radiation was the Pen-Ray lamp model 90-0019-04 with λ_max_ = 365 nm and an incident photon flux of *N*_p_ = 4.295 × 10^−6^ mol s^−1^ and the UV-C irradiation source was also the Pen-Ray lamp model 90-0004-07 with λ_max_ = 254/185 nm (UV-C lamp) and an incident photon flux of *N*_p_ = 1.033 × 10^−6^ mol s^−1^ (UVP, Upland, CA, USA). The incident photon flux was determined by actinometric experiments following the procedure described in [12]. The lamps were submerged in the investigated solution in the center of the reactor, so the UV radiation reached the wall of the reactor and induced the photocatalytic reaction. 

Reactor 2 was thermostated at 25 ± 0.2 °C. The glass ring with the catalyst was placed at the bottom of the reactor (Figure 1) with constant mixing and the distance between the LED lamp and reaction mixture was 20 cm for all experiments.

Samples for analysis were collected in defined time intervals and stored in the dark under 4 °C until analysis.

For all experiments, the rate constant (*k*) was determined by monitoring the decrease of azithromycin or sulfamethoxazole during the defined time intervals. The pseudo-first order kinetic model (ln(*c*_0_/*c*) = *k* ∙ *t*) was used for all experiments because it described the degradation of the chosen compounds under the investigated conditions well. 

### 2.4. Analytical Determination

Samples were collected in defined time intervals and analyzed on liquid chromatographs tandem mass spectrometers. An Agilent 1200 HPLC (Agilent Technologies, Waldbronn, Germany) system tandem triple quadrupole mass spectrometer (QQQ) Agilent 6410 was used to monitor the degradation rate of azithromycin and to propose degradation products whereas a Bruker amaZon ETD ion trap system coupled with an Ultimate 3000 RSLCnano system was used to monitor the degradation of azithromycin and sulfamethoxazole. 

The analytical procedure for analysis was on a high performance liquid chromatography (HPLC) Agilent Series 1200 HPLC system (San Diego, CA, USA) tandem triple quadrupole mass spectrometer Agilent 6410. The ionization was done with electrospray ionization (ESI) in positive mode. Newly formed compounds were separated on a Synergi Polar column (100 mm × 2.0 mm, 2.5 μm particle size) by Phenomenex (USA) and the volume of injection was 5 μL. The mobile phase used consisted of 0.1% formic acid in water (A) and in acetonitrile (B). Separation was obtained during gradient elution, which started with 8% of B, held for 3 min, and a linear increase of B during 12 min up to 95%, which was held for 5 min. The column was equilibrated during 10 min with 0% of B. Conditions for the mass spectrometer were: Drying gas temperature of 350 °C, capillary voltage of 4 kV, drying gas flow of 11 L min^−1^, and nebulizer pressure of 35 psi. Data were processed using Agilent MassHunter software version B.01.03.

Online LC-MS/MS analysis was performed on a Bruker amaZon ETD ion trap system (Bruker Daltonik GmbH, Bremen, Germany) coupled with an Ultimate 3000 RSLCnano system (Dionex, Amsterdam, The Netherlands). The sample injection, separation, and MS acquisition were carried out automatically. Solvents used for HPLC separation were methanol (solvent A) and 20 mmol/L ammonium formate (solvent B) at pH 4. Chromatographic separation was carried out on a Waters Acquity analytical column (Acquity UPLC HSS T3 50 mm × 2.1 mm i.d., 1.8 μm particle size, Waters, USA). Separation was achieved using the following 11 min gradient: 0.0–0.5 min, 30% B; 0.5–7 min, 30%–95% B; 7–9 min, 95% B; 9–11 min, 30% B. The mass spectrometer operated under unit resolution in the selected reaction monitoring (SRM) mode. Positive ionisation mode was used and the electrospray capillary voltage was set as −4500 V. The temperature and flow rate of the drying gas were set at 200 °C and 5 L/min, respectively. Helium was used as the collision gas. The following quantifier transitions were monitored in the SRM mode: (I) Sulfamethoxazole 254.1 > 156.5 m/z at the retention time of 2.9 min (isolation with 3 *m*/*z*, fragmentation amplitude 0.7 V), and (II) azithromycin 375.2 > 591.3 *m*/*z* at the retention time of 5.5 min (isolation with 3 *m*/*z*, fragmentation amplitude 0.7 V). For confirmation of the identity of the parent compound, whole tandem MS spectrum was considered. Sulfamethoxazole and azithromycin compounds were quantified using QuantAnalysis (Bruker Daltonik GmbH, Bremen, Germany) and the Bquant tool [13]. Matrix-matched calibration curves were constructed and limits of quantification (LOQ) were defined as the lowest point of the calibration curve with a signal-to-noise (S/N) ratio ≥10.

## 3. Results

### 3.1. Micro-Raman and AFM analysis of the Sol-Gel Nanostructured TiO_2_ Film

**Figure 2:**
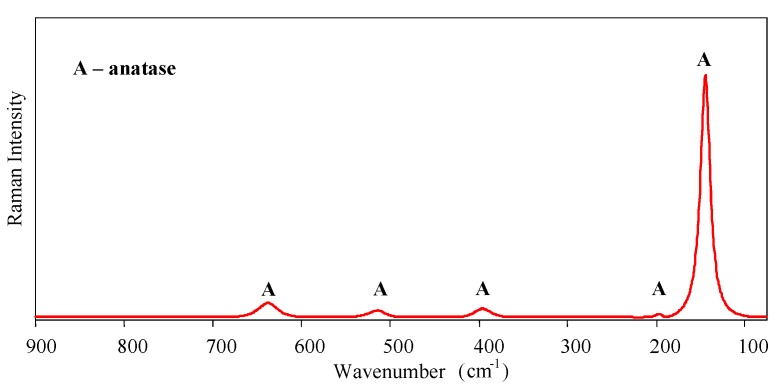
Raman spectra of the sol-gel nanostructured TiO_2_ film.

Micro-Raman spectroscopy was performed to identify the crystalline phases of the sol-gel nanostructured TiO_2_ film. Raman spectra are shown in Figure 2. Only the anatase phase was found in the film—the typical bands of the anatase phase appear around 143 (Eg), 196 (Eg), 396 (B1g), 514 (A1g, B1g), and 638 cm^−1^ (Eg).

**Figure 3:**
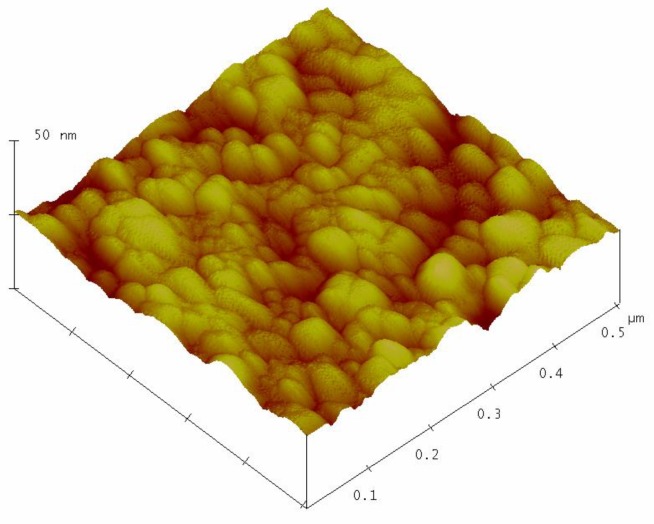
Surface topography of the sol-gel nanostructured TiO_2_ film.

Figure 3 shows the surface topography of the sol–gel nanostructured TiO_2_ film obtained by AFM analysis.

The AFM analysis of the sol-gel nanostructured TiO_2_ film on the glass substrate shows granular microstructures containing regular, almost monodispersed spherical particles. The mean grain size of the nanostructured TiO_2_ film obtained by AFM analysis was 28.3 ± 2.6 nm. 

### 3.2. Photolytic Degradation

**Figure 4:**
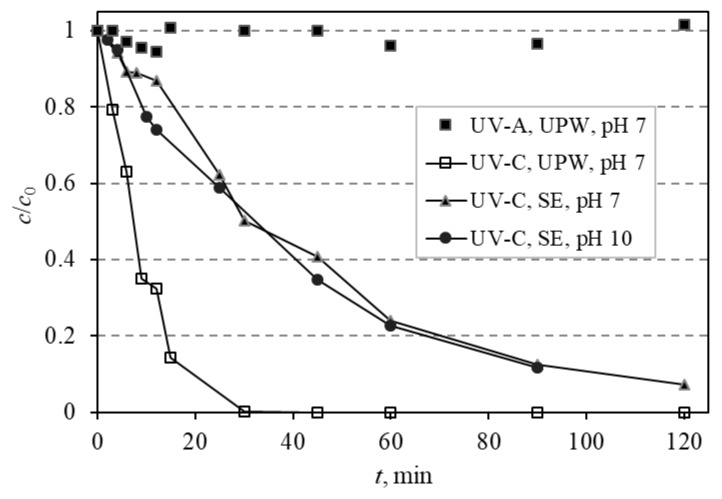
Photolytic degradation of azithromycin using UV-A and UV-C radiation at different pH (7 and 10) in different matrix (UWP—ultrapure water, SE—synthetic effluent).

Azithromycin was subjected to photolytic degradation using two different irradiation sources: UV-A and UV-C Pen-Ray lamps, and the effect the water matrix may have on the degradation rate was investigated. When UV-A (365 nm) radiation was used in ultrapure water (UPW) at a pH of 7, no degradation of azithromycin occurred. Conversely, under the irradiation of UV-C (254/185 nm), azithromycin was completely degraded during 30 min (Figure 4).

The influence that the water matrix and pH may have on the degradation rate using UV-C radiation was investigated. The matrix that was used for subsequent experiments was synthetic wastewater effluent (SE) whose pH was adjusted to 7 and 10 to compare the obtained degradation rates. Degradation of AZI was faster in UPW than in SE because part of the irradiation energy is being used on the degradation of components of SE instead of only on azithromycin. 

### 3.3. Photocatalytic Degradation

The main aim of this study was to investigate photocatalytic degradation of azithromycin and what influence different parameters (irradiation source, pH, matrix influence, influence of another compound) may have on the degradation rate. 

Firstly, photocatalytic experiments were done with the solution of azithromycin in ultrapure water at a pH of 7 on sol-gel TiO_2_ film with different irradiation sources: UV-A and UV-C. As expected, UV-C radiation proved to be more efficient in the photocatalytic degradation of AZI. Based on this data, the UV-C radiation was chosen for subsequent photocatalytic experiments with sol-gel TiO_2_ film. Also, experiments in the dark were done and there was no decrease in the azithromycin quantity during this experiment, showing that the degradation in subsequent experiments was due to photocatalysis.

The influence that the pH of the investigated solution has on the degradation process using selected experimental conditions (UV-C + TiO_2_) was investigated for three values: 3, 7, and 10. The pH of the solution governs several different actions and the impact it has on degradation is complex. In conclusion, the results showed that the favorable pH for azithromycin degradation was pH 7 and 10. These conditions were taken as optimal for azithromycin degradation so the following experiments were done under these conditions (UV-C + TiO_2_ film, pH 7 and 10). The obtained results provided information on the efficiency of photocatalytic degradation of AZI in ultrapure water and those results were used as the basis for the next experiments with SE. 

To investigate the influence of the matrix on the photocatalytic degradation of azithromycin, it was prepared in synthetic wastewater effluent (SE). The influence of pH was also investigated in SE so the pH of SE was adjusted to 7 and 10, as these pH are optimal for the degradation of AZI. The matrix itself had very little influence on the photocatalytic degradation most likely because AZI is primary in all degradation processes compared to other constituents. A pH of 10 was optimal for degradation in SE as it was also optimal for degradation in UPW (Figure 4). 

**Figure 5:**
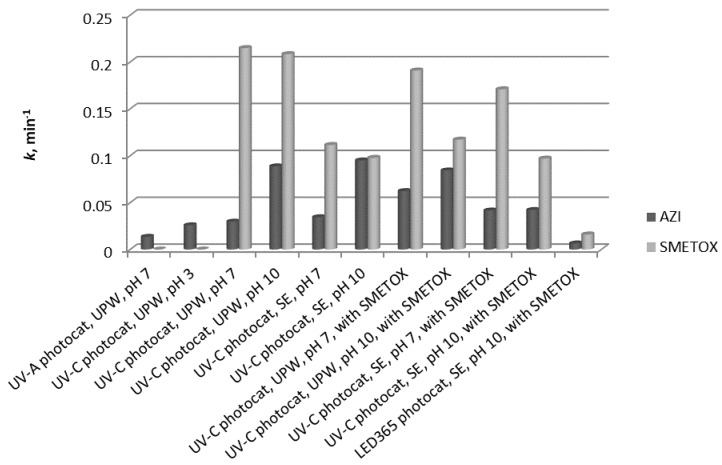
Degradation rate constants for azithromycin photocatalytic degradation under investigated conditions (UPW—ultrapure water; SE—synthetic effluent).

Photocatalysis of sulfamethoxazole was studied under different experimental conditions, such as pH and the matrix (SE and UPW), to gather information on its degradation before studying photocatalysis of the mixture of AZI and SMETOX. Degradation in ultrapure water was satisfactory at both pH because complete degradation was achieved in less than 45 min despite the possible drawbacks due to repulsion of the compound and the catalyst. There was a slight difference in the degradation rates in favor of pH 7, but this was not very distinguishable (Figure 5). Degradation of sulfamethoxazole was also studied in synthetic effluent under pH of 7 and 10 to examine if the components of effluent influence the degradation rate (Figure 5). By comparing the degradation of sulfamethoxazole in ultrapure water and in SE, it is evident that the degradation in SE is a little slower, most likely because the components of SE overtake the radical species, so they are being degraded instead of SMETOX. Also, the change in the pH of SE did not significantly influence the degradation of sulfamethoxazole although at pH 7, the degradation is a little faster.

Previous experiments showed the behavior of AZI and SMETOX during photocatalysis under different conditions: Different matrices and pH. The gathered data were used as the foundation for interpreting the behavior of these two pharmaceuticals during photocatalysis when they were together in a solution. Azithromycin and sulfamethoxazole were prepared as a mixture and the concentration of each compound was 10 mg/L. They were prepared both in ultrapure water and in SE, and the pH was adjusted to 7 and 10 for both matrices. Azithromycin was degraded better when the pH of the solution was 10 regardless of the matrix. In the experiments where the matrix was UPW at pH 7, the presence of sulfamethoxazole increased the degradation rate of azithromycin. Conversely, the presence of sulfamethoxazole negatively affected the degradation of azithromycin and the degradation rate was lower. Sulfamethoxazole was degraded faster in SE in the presence of azithromycin. 

In this research, an LED 365 nm lamp was used as the source of radiation, which should also activate the sol-gel TiO_2_ nanostructured film. Its efficacy was investigated for samples of azithromycin and sulfamethoxazole prepared in SE at pH 10, simulating real conditions. The degradation of azithromycin was slightly faster than degradation of sulfamethoxazole. 

### 3.4. Degradation Products of Azithromycin and their Toxicity

During the investigated photocatalytic degradation of azithromycin, five degradation products were identified. None of the reported degradation products (DPs) were identified in blanks or in standard solutions. There is a lack of research considering the degradation of macrolides, especially azithromycin, with advanced oxidation processes [14,15]. To our present knowledge, the photocatalytic degradation of azithromycin in simulated real conditions and degradation products formed after such degradation has not yet been reported. DPs were determined using HPLC–MS/MS and based on mass spectra and fragmentation pattern structural formulae were proposed (Table 1). DP4 and DP5 were the result of aminosugar cleavage from the cyclic lactone ring. DP5 is the result of cleavage of only the desosamine sugar from the lactone ring and DP4 of only the cladinose sugar. Their fragmentation shows the loss of the remaining sugar from the central ring. These degradation products were detected in previous studies dealing with photolytic degradation of macrolide antibiotic and azithromycin among others [16,17]. DP3 is the result of a loss of both amino sugars from the lactone ring and was also reported in previous photolytic studies [16,17]. DP2 presents an opening of the lactone ring after the loss of both sugars, together with *N*-demethylation and hydroxylation. DP1 is a result of further degradation of DP2 and the lactone ring. Degradation of azithromycin can be described as cleavage of the amino sugars from the cyclic lactone ring and further degradation of the lactone ring itself.

**Figure 6:**
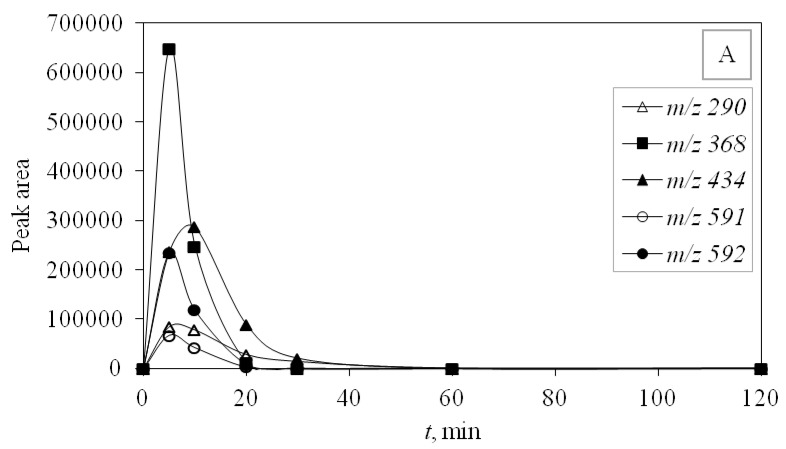

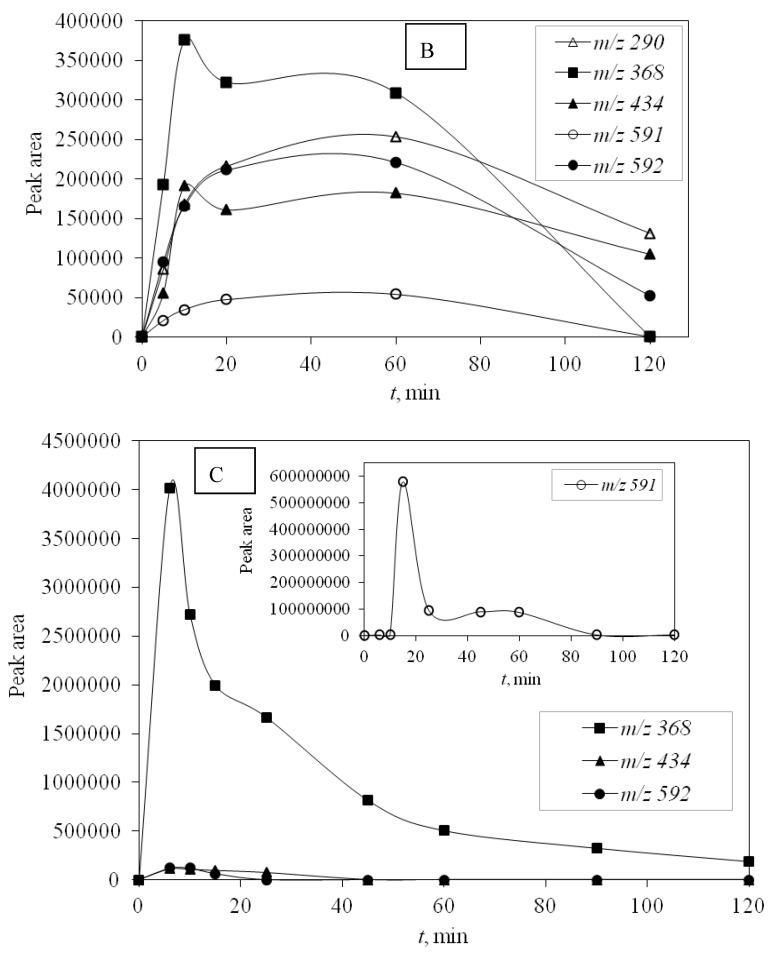
Profiles of degradation products at (**A**) UV-C + TiO_2_ UPW pH 7; (**B**) UV-A + TiO_2_ UPW pH 7; (**C**) LED 365 nm + TiO_2_ SE pH 10.

UV-C radiation also proved to be more effective in removing DP than UV-A, and for the same reason, it was more effective in removing the main compound—higher energy. DPs were monitored during photocatalytic degradation with LED 365 nm radiation under simulated real conditions (SE) adjusted for optimal degradation efficiency (pH 10) to identify the efficiency of promising LED photocatalysis. During this type of process, only four degradation products occurred and DP1 was not formed. Other degradation products were detected and they were successfully degraded during the investigated time of 120 min (Figure 6).

Toxicity was determined for the samples after photolytic and photocatalytic degradation, where the sources of radiation were UV-C and UV-A radiation. Toxicity was investigated using *Vibrio fischeri* and the toxicity was determined by measuring their luminescence inhibition, such that if the compounds in the sample were toxic, the luminescence would decrease. Samples were taken at defined time intervals during the degradation of azithromycin in ultrapure water with UV-C and UV-A radiation. The starting concentration of azithromycin was 10 mg/L and samples were taken after 5, 10, 20, 30, 60, and 120 min. None of the investigated samples showed any toxicity, which proves that the newly formed degradation products are not toxic. These results show that photocatalytic degradation is an efficient way of removing azithromycin from the waste waters without producing new compounds which are toxic.

## 4. Discussion

### 4.1. Photolytic Degradation

Photolytic degradation of azithromycin was investigated under various conditions. Complete degradation under UV-C (254/185 nm) irradiation within 30 min (Figure 4) in comparison to no degradation under UV-A (365 nm) irradiation was as expected, considering that UV-C irradiation has higher energy and therefore is more efficient in the degradation of organic compounds. Because of this finding, further experiments were conducted using this (UV-C) light source.

Photolysis was investigated under different matrices and pH. The change of pH did not have any influence on the degradation of azithromycin in synthetic effluent (Figure 4). Other research show that azithromycin is prone to photolytic degradation. Voight et al. [16] degraded azithromycin using polychromatic light and it was degraded after 10 min. The difference in results from this study is not very significant and can be explained by the different radiation source and reactor set-up. Azithromycin was also degraded using solar-like light and it was not completely degraded after 25 h [17], showing that to degrade completely complex compounds, such as azithromycin, radiation with higher energy should be used.

### 4.2. Photocatalytic Degradation

All photocatalytic experiments in this research were done using sol-gel nanostructured TiO_2_ film because of its many advantages. Firstly, titania has been proven to be an efficient photocatalyst for different pollutants’ degradation. In most studies, it is used in the form of a suspension, which allows an efficient reaction, but the main disadvantage is its difficult removal from the reaction mixture after the process is over. The catalyst is usually removed by filtration, which adds an extra step to the whole process. Therefore, its applicability in this form is not optimal for applications in real systems. These drawbacks of the application of TiO_2_ in the suspension form may be resolved by immobilization of TiO_2_ on different substrates in the form of a thin nanostructured ceramic film [18,19,20], which was applied in this work. The most widely used method for producing this film is the sol-gel synthesis process [21].

To explain the results gathered during photocatalysis under different pH, it is necessary to take into consideration some characteristics of titania under different pH. Isoelectric point (IEP) values have been reported in the literature for anatase for TiO_2_ ranging from 5.1 to 6.7 [22,23], which means that at pH lower than that value, the surface becomes positively charged, and at pH higher than 6.7, the surface is negatively charged. The dissociation constant for azithromycin is 8.96 [24], so in solutions with a pH lower than that, azithromycin is in the cationic form, and in solutions with a higher pH, it is in its neutral form. Ionic distribution at different pH can lead to an explanation about AZI’s behavior during photocatalysis at different pH. The fastest degradation was achieved at pH 10, followed by degradation at pH 7, and the slowest degradation was at pH 3 (Figure 5). At pH 10, the surface of the catalyst is negatively charged and azithromycin is in its neutral form. There are no repulsive forces and the degradation can progress. On the other hand, at pH 3, the surfaces of both the catalyst and azithromycin are positively charged so repulsive forces are negatively influencing the degradation. Additionally, at pH 10, there are more OH^−^ species, which makes the formation of OH• easier. This is important because hydroxyl radicals are, in most cases, species that are mainly responsible for degradation and therefore their larger number increases the degradation rate. Also, under alkaline conditions, the valence band and holes can react with water and generate hydroxyl radicals [24]. In conclusion, the results showed that the most favorable pH values for azithromycin degradation are pH 7 and 10. Photocatalysis of sulfamethoxazole was also investigated under different pH values of the solution. At pH 7 and 10, it is in its negative ionic form and there is possible repulsion between the molecules of sulfamethoxazole and the surface of the catalyst [19] because sulfamethoxazole has two dissociation constants, p*K*_a1_ = 1.86 and p*K*_a2_ = 6.04 [24,25].

Within photocatalytic experiments, different irradiation sources were tested and one of them was the experiment with the LED 365 nm radiation source because of an increasing interest in using LED lamps for photocatalytic purposes considering their many advantages [20]. LED lamps yield a better efficiency in converting electrical energy to light, meaning higher quantum yields; they are small, compact, and robust. They do not require any warm-up time and they have a long life time. Also, one of the meaningful advantages is that they do not generate mercury waste and therefore there is no necessity for special disposal of used lamps [20,26]. Photocatalytic degradation using LED lamps applied for pharmaceutical removal under described conditions was satisfactory compared to previous research studying pharmaceutical degradation with LED lamps [27,28,29,30]. Conversely, compared to experiments with the same conditions (SE and pH 10), which included an Hg based 254 nm lamp, the degradation of both compounds was slower. Irradiation of 254 nm has higher energy than 365 nm, but despite this property, this research showed that the LED 365 nm lamp is still efficient for photocatalytic degradation of complex compounds, such as pharmaceuticals. 

## 5. Conclusions

The purpose of this research was to investigate the photocatalytic degradation of azithromycin under real life conditions to prove that photocatalytic oxidation is an option for waste water purification. For the photocatalytic experiments, nanostructured TiO_2_ film deposited on a borosilicate glass substrate by the by sol-gel process and dip-coating technique was used. 

The important findings of the current study can be summarized as follows:

− TiO_2_ film consisted of the TiO_2_ anatase phase and showed a granular microstructure, with almost monodispersed spherical particles with a grain size of 28.3 ± 2.6 nm.
− Optimal photocatalytic degradation of azithromycin was achieved using UV-C radiation with sol-gel TiO_2_ film as a catalyst at pH 10.
− Photocatalytic degradation of azithromycin was not influenced by the presence of sulfamethoxazole.
− Azithromycin was successfully removed from simulated real waste water by photocatalysis using TiO_2_ film and different irradiation sources.
− An LED 365 nm lamp proved to be efficient in photocatalytic degradation.
− Five degradation products were detected; they have not been previously reported and none of the compounds were toxic.

## Figures and Tables

**Table 1:** Degradation products of azithromycin with their *m/z* values, proposed structures, fragments, and fragmentation energies.

**Name Molecular Formula *m*/*z***	**Structural Formula**	**Fragment (Fragmentation Energies)**
**Azithromycin** C_38_H_72_N_2_O_12_ *m/z* 749	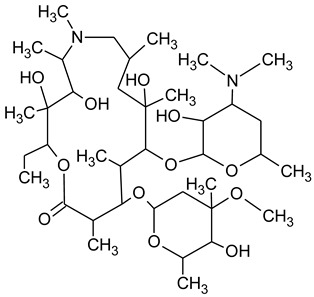	591 (20 eV)
**Name Molecular Formulae *m*/*z***	**Proposed Structural Formulae**	**Fragments (Fragmentation Energies)**
**DP1** C_15_H_31_NO_4_ *m/z* 290	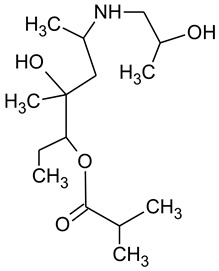	129 57 (20 eV)
**DP2** C_16_H_33_NO_8_ *m/z* 368	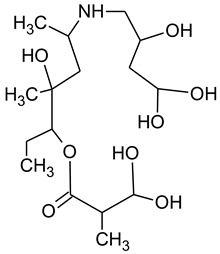	336 350 (20 eV)
**DP3** C_22_H_43_NO_7_ *m/z* 434	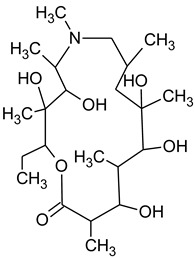	416 274 155 (25 eV)
**DP4** C_30_H_58_N_2_O_9_ *m/z* 592	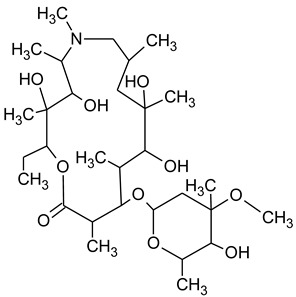	116 158 (25 eV)
**DP5** C_30_H_58_N_2_O_9_ *m/z* 591	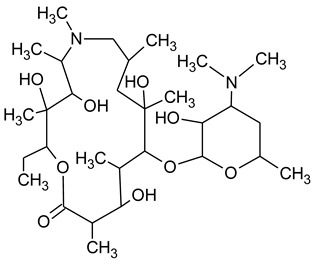	158 416 (25 eV)
